# Exploring University Faculty’s AI Well-Being: A Structural Equation Model of Social Supports, AI Literacy, and Technological Self-Efficacy

**DOI:** 10.3390/bs16071168

**Published:** 2026-07-10

**Authors:** Weitong Liu, Yukun Li, Yuxuan Yan, Jiahan Wang, Jinyu Wang, Hui Zhang

**Affiliations:** 1 School of International Education, Shandong University, Jinan 250100, China; lwt@sdu.edu.cn (W.L.); liyukun202422360014@mail.sdu.edu.cn (Y.L.); risataaaa@163.com (Y.Y.); wjh202320073016@mail.sdu.edu.cn (J.W.); 2 Institute for Advanced Studies in Education, Shandong University, Jinan 250100, China; 3 Department of Higher Education Administration, National Academy of Education Administration, Beijing 102617, China

**Keywords:** AI well-being, AI literacy, technological self-efficacy, social support, organizational support, higher education

## Abstract

As artificial intelligence (AI) technologies become increasingly embedded in higher education, concerns have emerged regarding their psychological impact on university faculty. While existing research has largely focused on technological readiness and digital competencies, the social–psychological foundations of faculty well-being in AI-integrated teaching environments remain insufficiently explored. Drawing on social support theory and self-determination theory, this study proposes and tests a structural model of AI-related well-being among university faculty. A total of 523 faculty members in China participated in a cross-sectional survey measuring perceived social support, organizational support, AI literacy, technological self-efficacy, and AI well-being. Structural equation modeling (SEM) was used to examine the hypothesized pathways and mediating mechanisms. The results indicate that both social support and organizational support significantly and positively influence AI literacy and technological self-efficacy. In turn, AI literacy and technological self-efficacy significantly predict AI well-being and serve as key mediators. Among all factors, AI literacy exhibits the strongest total effect on AI well-being, followed by social support, organizational support, and technological self-efficacy. This study contributes to the theoretical understanding of faculty psychological adjustment during technological transitions and offers practical recommendations for institutions seeking to cultivate AI-ready and psychologically supportive academic environments.

## 1. Introduction

The rapid integration of artificial intelligence (AI) into educational systems has fundamentally reshaped the landscape of teaching, learning, and institutional operations in higher education. AI applications, including generative AI tools, intelligent tutoring systems, automated grading, lesson planning, and predictive learning analytics, are increasingly adopted to enhance instructional efficiency and personalize the learning experience. (Huang et al., 2025; J. Kim, 2024; Luckin et al., 2016; UNESCO, 2021; Zhai, 2023). Far from serving a singular role, these technologies now function across multiple domains—as virtual instructors, teaching assistants, administrative aides, and even instructional designers (Alam, 2021; Whalen & Mouza, 2023). By taking on these supportive roles, AI can help users establish a sense of teaching control and autonomy, laying a foundation for their well-being (Huang et al., 2024).

As AI technologies become increasingly integrated into daily teaching practices, the role of teachers is undergoing a fundamental shift—from passive transmitters of knowledge to active co-designers of learning experiences in collaboration with intelligent systems (Taufikin et al., 2024). While this transformation offers clear educational benefits, it also imposes significant psychological, cognitive, and emotional demands on educators (Zhai, 2025). For example, AI-driven tools designed to ease workload, such as automated assessment systems, may inadvertently amplify social biases and faculty stress (Cheuk, 2021). Moreover, inadequate technical training and limited access to professional development contribute to heightened technology-related anxiety (Yang & Wang, 2024).

This complex dynamic of change reveals a deeper paradox in AI integration: while generative AI can enhance both instructional efficiency and learners’ self-efficacy, thereby contributing to enhancing well-being (Fan & Cui, 2024), its excessive use may undermine human-centered pedagogy, reducing authentic interpersonal interaction, particularly in teacher education programs (J. Zhang & Zhang, 2024). For educators, this duality manifests as both empowerment through innovative tools and disempowerment through diminished autonomy and increased psychological vulnerability (L. Zhang & Xu, 2025), which makes it difficult for self-efficacy to be translated into genuine well-being. It is also reshaping people’s perceptions of education (Huang et al., 2024), leading teachers to develop a “love-hate relationship” with AI (Toncelli & Kostka, 2024). Such contradictions have prompted educators to reconsider how technological innovation can coexist with traditional pedagogical values, often triggering tension and reconfiguration of professional identity (Lan, 2024). These fluctuations in professional identity constitute the core psychological dimension that influences teachers’ well-being.

Despite growing scholarly attention to AI adoption, most empirical studies in higher education remain concentrated on external factors such as technological readiness, perceived usefulness, and attitudes toward AI, typically guided by frameworks like the Technology Acceptance Model (TAM) (Teo et al., 2009; Venkatesh & Davis, 2000). While these perspectives yield critical insights, they often neglect the deeper socio-psychological processes—emotional, cognitive, and motivational—that fundamentally shape learners’ and educators’ well-being in AI-integrated environments (Huang et al., 2025; Huang & Zou, 2024). In particular, affective dimensions such as teachers’ enthusiasm, self-efficacy, and emotional responsiveness to AI tools play a crucial mediating role in technology adoption and occupational well-being, yet remain under-theorized within dominant technology acceptance paradigms (Huang et al., 2024).

To address these gaps, this study shifts the research focus from technology adoption to socio-psychological mechanisms shaping faculty well-being during AI integration. Drawing on social support theory (Cohen & Wills, 1985) and self-determination theory (Deci & Ryan, 2000), this study develops and tests a structural model linking social support, organizational support, AI literacy, technological self-efficacy, and AI well-being among university faculty in China. The integration of these two theoretical perspectives offers a comprehensive explanatory framework: social support theory emphasizes the external and contextual resources—such as collegial, institutional, and organizational support—that buffer stress and foster adaptation, while self-determination theory highlights internal motivational processes driven by the fulfillment of autonomy, competence, and relatedness needs. Together, they illuminate how social and organizational supports interact with individual competencies (e.g., AI literacy, self-efficacy) to promote faculty’s psychological well-being amid technological change.

By emphasizing the interplay between individual capability and contextual support, this study contributes to a deeper theoretical understanding of faculty psychological adjustment during technological transitions. Unlike technology acceptance research that primarily explains whether educators intend to use or adopt AI, this study focuses on how educators psychologically adapt to AI integration and experience well-being in AI-supported professional contexts. It further positions AI literacy as a key mechanism through which social and organizational supports are translated into positive psychological outcomes, because AI literacy enables faculty not only to use AI tools but also to understand, evaluate, and pedagogically integrate them in meaningful and responsible ways. In this sense, the study extends existing work on technology acceptance, teacher self-efficacy, and digital well-being by proposing a support–literacy–well-being pathway for understanding faculty adaptation to AI in higher education.

## 2. Literature Review

### 2.1. AI Well-Being in Higher Education

Psychological well-being, traditionally defined as sustained happiness, mental health, and life satisfaction (Huppert, 2009), involves a dynamic balance between positive and negative emotional states and reflects one’s capacity for self-realization and purposeful engagement (Ryff, 1989). More broadly, teacher well-being is a well-established predictor of professional commitment, instructional quality, and student outcomes. Teachers with high well-being demonstrate better emotional regulation, stronger student relationships, and higher instructional engagement (Hascher et al., 2021; Roffey, 2012). Longitudinal findings further support that teacher well-being is positively associated with self-efficacy and instructional enthusiasm. Conversely, low well-being contributes to burnout, absenteeism, and turnover, undermining both faculty performance and institutional stability (Dreer, 2023).

In the present study, AI well-being refers specifically to faculty members’ positive psychological functioning and adaptive experiences when engaging with AI-supported teaching and academic work. It is not intended to represent general teacher well-being, overall job satisfaction, digital well-being, or the mere absence of technostress. Rather, it captures the extent to which faculty experience positive emotion, engagement, supportive relationships, meaning, and accomplishment in relation to AI use in higher education.

As technology becomes deeply embedded in higher education, researchers have begun to examine how digital transformation affects teachers’ well-being. Faculty with access to streamlined workflows often report greater motivation and engagement in creative teaching practices, which reinforce their sense of professional competence and fulfillment (Peccei, 2004). Technology acceptance factors—particularly perceived usefulness and ease of use—have also been linked to reduced stress and enhanced job satisfaction (Davis et al., 1989). Moving beyond these foundational technologies, recent empirical work indicates that the adoption of generative AI can enhance subjective well-being by increasing feelings of efficacy, happiness, energy, and satisfaction, while simultaneously mitigating stress and anxiety (Cambra-Fierro et al., 2025).

However, these benefits are accompanied by complex trade-offs (Sharma, 2024). Critical scholarship urges caution against technological optimism, pointing out that overreliance on AI may exacerbate existing ethical tensions around academic integrity, data privacy, and—more fundamentally—the progressive erosion of professional autonomy (Alam, 2021; Li et al., 2024). Such concerns are heightened by emerging evidence that both teachers and students express ambivalence about whether generative AI might eventually displace core instructional roles, thereby altering educator identity and agency (Chan & Tsi, 2024; Whalen & Mouza, 2023).

This dual nature of AI adoption resonates with Peccei’s (2004) perspective that well-being emerges from navigating tensions between gains (e.g., efficiency, personalization) and risks (e.g., ethical dilemmas, technostress). As such, AI-driven well-being is inherently multidimensional and requires both adaptive responsibility and ethical vigilance. Current literature remains notably sparse in capturing how these dynamics concretely manifest within real-world AI-integrated educational settings, particularly from a faculty psychological perspective.

### 2.2. Social and Organizational Support

Social and organizational support represent two key dimensions of external resources that shape educators’ adaptation and well-being in AI-mediated higher education. Rooted in perceived organizational support theory (Eisenberger et al., 1986), these supports are conceptualized as institutional and interpersonal mechanisms that enhance teachers’ motivation, competence, and psychological resilience. Social support refers to interpersonal assistance and collegial collaboration through professional networks or communities of practice, while organizational support encompasses the structural and policy-level provisions—such as infrastructure, leadership, and training—that facilitate technology integration (Inan & Lowther, 2010; Liu & Hu, 2025; Venkatesh & Davis, 2000). Organizational support is a key factor influencing teachers’ adoption of AI, as it can moderate the impact of other factors (such as compatibility and trialability) on adoption behavior. Strong institutional support, practical opportunities, and training are crucial for promoting effective adoption (Nagae et al., 2025; C. B. Zhang et al., 2024).

Within collegial settings, peer collaboration and community interaction strengthen emotional resilience and technological readiness by fostering trust, shared knowledge, and a sense of belonging (Trust & Prestridge, 2021). Such peer networks encourage pedagogical experimentation, develop digital fluency, and reduce feelings of isolation, which together contribute to teachers’ emotional well-being. Social support also helps to regulate the user-AI assistant relationship, leading to the establishment of a partnership, which has been proven to enhance the subjective well-being of users (Aldraiweesh & Alturki, 2025; C. B. Zhang et al., 2024).

Organizationally, access to technological infrastructure, technical assistance, and professional development opportunities enhances educators’ perceived control and efficacy in adopting AI tools (Fu, 2025). Institutional strategies that integrate emotional and mental health support are also associated with lower technostress and higher professional satisfaction. Empirical studies have shown that the intelligent teaching support provided by higher education institutions is beneficial for enhancing teachers’ information technology expertise, thereby leading to better teaching outcomes (Cao et al., 2026; S. Wang et al., 2021).

Overall, while both forms of support help mitigate stress, organizational support offers structural assurance and resource stability, whereas social support primarily strengthens emotional resilience and peer connectedness. Their interaction produces a combining effect that jointly enhances faculty adaptation to AI technologies. However, existing research has often examined these two constructs in isolation, overlooking their dynamic interdependence and complementary roles in shaping teachers’ psychological well-being. This study argues that it is precisely through their complementary roles in fulfilling psychological needs for relatedness and competence that these supports influence well-being, thereby forming the theoretical basis for the hypothesized pathways in the present model.

### 2.3. AI Literacy and Technological Self-Efficacy

In today’s digital and knowledge-based society, AI literacy is rapidly emerging as a foundational skill for educational professionals. AI literacy involves a range of cognitive, technical, and reflective capabilities that enable individuals to evaluate, communicate, and collaborate with AI tools in diverse contexts (Long & Magerko, 2020; Tseng & Warschauer, 2023). According to UNESCO’s (2024) latest report, these capabilities of teachers include understanding, acquisition, and navigation, prompting, verification, and integration, which together promote both critical and practical engagement with AI technologies.

Empirical research increasingly highlights the pivotal role of AI literacy in shaping educators’ motivation, confidence, and well-being in AI-enhanced teaching environments. High levels of AI literacy are associated with stronger perceptions of AI as a tool for social good, greater self-efficacy in learning and applying AI, and heightened awareness of ethical considerations in educational use (Du et al., 2024). Enhanced AI literacy enables educators to streamline instructional design and administrative tasks through AI-assisted tools, thereby alleviating workload stress and promoting teaching well-being (Velander et al., 2024). Moreover, AI-literate teachers are better equipped to guide students in the responsible and critical use of generative AI tools, integrating reflective thinking into AI-supported learning activities such as writing courses (Sherwood & Mac Donald, 2024). Teachers with higher levels of AI literacy can better utilize AI to identify individual differences and make real-time adjustments to teaching, enhancing the inclusiveness and fairness of education (Daher, 2025). Overall, AI literacy functions as both a cognitive and psychosocial enabler that strengthens educators’ autonomy, motivation, and sense of professional purpose.

Technological self-efficacy has become a critical construct in understanding educators’ psychological and behavioral adaptation to technology-rich teaching environments. It refers to individuals’ confidence in their ability to effectively perform tasks involving digital or automated systems (McDonald & Siegall, 1992). Higher levels of technological self-efficacy are associated with stronger trust in technology, more positive attitudes toward AI systems, and greater self-assurance when interacting with intelligent tools (Montag et al., 2023). In educational contexts, teachers with high technological self-efficacy demonstrate greater willingness to adopt innovations, stronger problem-solving persistence, and more effective strategies for overcoming technical challenges (Teo et al., 2009). Continuous exposure to emerging technologies further reinforces such confidence, indicating that technological proficiency and self-efficacy mutually strengthen one another over time (Berdousis, 2024).

Empirical evidence consistently shows that technological self-efficacy significantly enhances teachers’ capacity to integrate technology into instruction. Educators with high self-efficacy are more likely to employ innovative pedagogical approaches, align digital tools with curricular goals, and sustain enthusiasm for teaching (Czerniak & Lumpe, 1996; Gomez et al., 2022). The adoption of such proactive technologies and the application of innovative teaching methods can not only enhance teaching effectiveness but also boost teachers’ confidence in their own teaching competencies, thereby fostering their professional satisfaction (Paetsch et al., 2023; Vieira et al., 2024). Elevated self-efficacy also mitigates technostress, reduces burnout, and alleviates AI-related anxiety by fostering a greater sense of control over technological tools (B.-J. Kim & Kim, 2024; M. Wang et al., 2024). Such evidence underscores that technological self-efficacy serves as a crucial psychological resource that empowers educators to navigate and thrive in AI-integrated teaching environments.

### 2.4. Theoretical Framework and the Present Study

As AI technologies increasingly permeate higher education, university faculty face growing cognitive and emotional demands that extend beyond the acquisition of technical skills. While previous studies have examined technology acceptance and digital competence, less attention has been given to the psychological well-being of educators in AI-mediated environments. To address this gap, the present study integrates perspectives from social support theory and self-determination theory to construct a comprehensive framework for understanding AI-related well-being among university faculty.

According to social support theory, supportive relationships and institutional structures provide external resources that buffer stress and enhance individuals’ ability to cope with professional challenges (Cohen & Wills, 1985). Self-determination theory further explains that human well-being depends on the satisfaction of three basic psychological needs—autonomy, competence, and relatedness (Deci & Ryan, 2000). Within higher education, social and organizational support can fulfill these needs by fostering belonging, providing growth opportunities, and enabling professional autonomy. Integrating these perspectives, external support (social and organizational) strengthens internal psychological resources (AI literacy and technological self-efficacy), which in turn promote educators’ AI-related well-being by satisfying both competence and relatedness needs. This multilevel mechanism reflects the dynamic interplay between contextual enablers and personal agency.

We propose that social and organizational support foster AI well-being precisely by nurturing these psychological needs, with AI literacy and technological self-efficacy serving as key mediators in this process. Specifically, supportive environments fulfill relatedness and build competence through knowledge sharing and skill development. Enhanced AI literacy and self-efficacy, in turn, empower educators to make autonomous and informed decisions about AI use. Thus, we propose a sequential psychological model: External Support → Internal Capacities (AI literacy/self-efficacy) → AI Well-being, which integrates social support theory’s ‘external resource buffering’ perspective with self-determination theory’s ‘internal need satisfaction’ mechanism. It elucidates how contextual supports enhance well-being by fostering the internal resources that enable autonomous practice. Building on this framework, we empirically test a structural equation model where AI literacy and technological self-efficacy mediate the effects of social and organizational support on AI well-being. Accordingly, the following hypotheses are formulated:

**H1.**  *Perceived social support positively predicts AI literacy.*

**H2.**  *Perceived social support positively predicts technological self-efficacy.*

**H3.**  *Organizational support positively predicts AI literacy.*

**H4.**  *Organizational support positively predicts technological self-efficacy.*

**H5.**  *AI literacy positively predicts AI well-being.*

**H6.**  *Technological self-efficacy positively predicts AI well-being.*

## 3. Methods

### 3.1. Participants and Data Collection

This study utilized data collected through a survey conducted in January 2025 at a high-level research-oriented university in China. This study was conducted at a high-level, research-oriented university located in an urban setting in Beijing, China. As a “Double First-Class” and “Project 211” university (i.e., designations that signify its status among the nation’s elite comprehensive, research-oriented institutions), the university hosts a large academic community with 25 departments, approximately 3200 full-time faculty members, and over 40,000 students across multiple campuses. Currently, the institution implements an active AI-integration framework that encourages the ethical use of Generative AI tools in hybrid teaching, scientific research, and administrative tasks. Faculty members are provided with institutional access to computational resources, and the university frequently hosts official workshops on Computer-Assisted Language Learning and AI literacy to enhance instructors’ technological self-efficacy (https://news.ncepu.edu.cn/xysx/37d75a4cb64f41768ee1b9e0e5430779.htm, accessed on 10 June 2026). This highly digitalized environment provides an ideal context for examining how generative AI affects faculty well-being and pedagogical practices. Participants were selected using a stratified random sampling approach to ensure diversity across academic disciplines and professional ranks. Faculty members from multiple schools (e.g., engineering, social sciences, humanities, and natural sciences) were invited via departmental announcements. Participation was entirely voluntary, and no monetary incentives were provided. The authors acted solely as external researchers during the data collection process. None of the authors were employed as instructors, administrators, or researchers at the university from which the participants were recruited. Therefore, the authors had no direct supervisory, instructional, or evaluative relationship with the participants.

The questionnaire was administered online using the Wenjuanxing platform (https://www.wjx.cn, accessed on 1 January 2025), a secure and widely used data collection tool in China. A statement at the beginning of the survey explicitly informed participants that they could choose not to answer any question and that completing and submitting the questionnaire constituted their informed consent to participate. No identifiable data was collected, and participants had the right to withdraw from the study at any time without any consequences. We affirm that this research adheres to ethical principles. A total of 523 responses were received, of which 502 were deemed valid after screening for missing or invalid entries, yielding a valid response rate of 96%.

The final sample consisted of faculty members from a wide range of academic disciplines, professional titles, and age groups. As shown in Table 1, the gender distribution was relatively balanced, with 46.22% male and 53.78% female participants. The age of respondents ranged from under 30 to over 56 years, with the largest proportions falling within the 31–40 age range. In terms of disciplinary background, 51.79% of participants were from engineering sciences, 27.29% from social sciences, 16.53% from the humanities and arts, and 4.38% from the natural sciences. Regarding professional rank, 56.97% held intermediate titles (e.g., lecturer, assistant professor), 30.88% held associate-level positions, and 12.15% were full professors or senior researchers. Overall, the sample demonstrated a high degree of diversity and representativeness, reflecting the demographic and academic characteristics of contemporary university faculty in China.

### 3.2. Instruments

The survey instrument consisted of five key constructs: social support, organizational support, AI literacy, technological self-efficacy, and AI well-being. Each construct was measured using a five-point Likert scale ranging from 1 (“strongly disagree”) to 5 (“strongly agree”). All measurement items were adapted from well-established instruments and localized based on prior literature. To ensure content validity and contextual appropriateness, two experts in the field were invited to review the questionnaire and provide suggestions for revision. In addition, two university faculty members participated in a pilot test and provided feedback on item clarity, wording, and overall comprehensibility. Based on their comments, minor revisions were made to improve the accuracy and readability of the questionnaire before formal data collection.

Social and Organizational Support. Drawing on diffusion of innovation theory (Rogers, 2003), environmental factors were operationalized through two dimensions: social support and organizational support. Social support included three subdimensions: colleague support, peer influence, and leadership influence. A total of eight items were used (e.g., “I can obtain help and advice from colleagues regarding AI-assisted teaching” and “The leadership of my department/college strongly supports my involvement in AI-innovative teaching”). The scale demonstrated excellent psychometric properties (KMO = 0.837; Cronbach’s α = 0.951). Organizational support comprised three subdimensions: strategic orientation, institutional incentives, and institutional guarantees. The scale included seven items (e.g., “The university has a clear strategic plan to promote AI-innovative teaching” and “The university’s network infrastructure and technical platforms are sufficient to support teachers in utilizing AI technologies.”) and showed high reliability and validity (KMO = 0.837; Cronbach’s α = 0.960).

AI Literacy. This study developed a localized AI literacy scale based on UNESCO’s 2024 AI Competency Framework for Teachers (UNESCO, 2024). The scale encompassed five core aspects: Human-Centered Mindset, Ethics of AI, AI Foundations and Applications, AI Pedagogy, and AI for Professional Development. Each aspect was assessed at three developmental levels—Acquire, Deepen, and Create—resulting in a total of 15 questions, such as “Recognizing that AI tools are human-driven” and “Effectively applying AI in instructional design and implementation.” Responses were recorded on a five-point Likert scale. The instrument exhibited strong internal consistency and construct validity (KMO = 0.948; Cronbach’s α = 0.953).

Technological Self-Efficacy. Technological self-efficacy was defined as instructors’ perceived ability to effectively integrate and use technology in their teaching. This construct builds upon the Technology Acceptance Model (Venkatesh & Davis, 2000), the Model of PC Utilization (Thompson et al., 1991), and Diffusion of Innovation Theory (Rogers, 2003), which emphasize the role of perceived usefulness and ease of use in shaping technology adoption. A four-item scale was used (e.g., “If I encounter information technology problems, I can generally resolve them myself” and “I am fully capable of independently using information technology to complete my daily work.”) to measure technological self-efficacy, yielding high reliability and construct validity (KMO = 0.843; Cronbach’s α = 0.921).

AI Well-Being. The construct of AI well-being was developed based on Seligman’s PERMA model—Positive Emotion, Engagement, Relationships, Meaning, and Accomplishment (Seligman, 2011)—as well as frameworks for psychological well-being (Ryff & Keyes, 1995) and teacher well-being (Stiglbauer et al., 2019). PERMA was selected because it provides a multidimensional framework for capturing positive psychological functioning rather than merely the reduction in negative states such as stress or anxiety. This is particularly relevant to AI-integrated higher education, where faculty well-being involves not only coping with technological demands but also experiencing positive emotion, engagement, meaningful professional use, supportive interaction, and accomplishment through AI-supported work. The scale aimed to holistically capture faculty members’ psychological states within AI-integrated teaching environments. It included five items, each corresponding to one dimension of the PERMA framework, such as “Using AI makes me feel happy and satisfied in my work” and “AI assists my work, giving me a strong sense of achievement.” The scale demonstrated excellent psychometric properties (KMO = 0.917; Cronbach’s α = 0.966). A complete list of the survey items for all constructs is provided in the Supplementary Table S1.

### 3.3. Data Analysis

To examine the proposed conceptual framework and test the hypothesized relationships, this study employed Structural Equation Modeling (SEM) using AMOS 26.0. We adopted SEM because the study involves multiple interrelated latent constructs and hypothesized indirect effects; SEM enables the simultaneous estimation of measurement and structural components while accounting for measurement error, providing advantages over separate regression models for testing complex mediation. The sample size (N = 502) is adequate for Maximum Likelihood (ML)-based SEM and bootstrapping, satisfying common guidelines for model estimation and stability. Model evaluation followed a systematic three-step procedure: (1) measurement model assessment, (2) structural model evaluation, and (3) mediation analysis. This multi-step approach allowed for a rigorous validation of both the measurement properties of the constructs and the structural pathways among variables.

Step 1: Measurement Model Assessment. The first step involved assessing the reliability and validity of the five latent constructs: social support, organizational support, AI literacy, technological self-efficacy, and AI well-being. Reliability and convergent validity were examined using the Kaiser–Meyer–Olkin (KMO) measure of sampling adequacy, Cronbach’s alpha (α), Composite Reliability (CR), and Average Variance Extracted (AVE). Acceptable thresholds were α and CR > 0.70 and AVE > 0.50, indicating satisfactory internal consistency and convergent validity.

Step 2: Structural Model Evaluation. The second step tested the overall model fit and the hypothesized directional relationships (H1–H6). Model fit was assessed using multiple indices: Chi-square to degrees of freedom ratio (χ^2^/df), Goodness-of-Fit Index (GFI), Normed Fit Index (NFI), Incremental Fit Index (IFI), Tucker–Lewis Index (TLI), Comparative Fit Index (CFI), and Root Mean Square Error of Approximation (RMSEA). Acceptable fit was indicated by GFI, CFI, TLI, NFI, and IFI > 0.90, χ^2^/df < 3, and RMSEA < 0.08. To enhance robustness, parameter estimates and standard errors were also validated using bias-corrected bootstrapping (2000 resamples). Standardized path coefficients and significance levels (*p*-values) were reported to determine support for the hypotheses.

Step 3: Mediation Analysis. The final step examined indirect pathways linking external supports (social and organizational) to AI well-being through internal capacities (AI literacy and technological self-efficacy). A bias-corrected bootstrapping procedure with 2000 resamples and 95% confidence intervals was employed to estimate direct, indirect, and total effects. Mediation effects were considered significant when the confidence interval did not include zero. This approach provided robust evidence for the mediating roles of individual-level competencies in translating external support into enhanced psychological well-being within AI-integrated teaching environments.

## 4. Results

### 4.1. Measurement Model Assessment

To ensure the reliability and validity of the measurement model, we conducted a series of confirmatory analyses on all five constructs: social support, organizational support, AI literacy, technological self-efficacy, and AI well-being. The results are presented in Table 2. All constructs demonstrated strong psychometric properties. The KMO values ranged from 0.837 to 0.948, indicating adequate sampling adequacy for factor analysis. Cronbach’s α values for all constructs exceeded the 0.90 threshold, ranging from 0.921 to 0.966, demonstrating excellent internal consistency reliability. C.R. values also met the recommended criterion of 0.70 or higher, ranging from 0.881 to 0.966, indicating satisfactory internal construct consistency. In addition, all constructs achieved AVE values above the 0.50 benchmark, ranging from 0.644 to 0.851, confirming convergent validity.

Specifically, the AI well-being construct achieved the highest AVE (0.851) and C.R. (0.966), suggesting a particularly coherent and reliable measurement of psychological well-being in AI-supported teaching environments. However, the very high Cronbach’s α value for this five-item scale may also indicate potential item redundancy, as some items may be conceptually close in capturing positive psychological experiences related to AI use. Therefore, this result should be interpreted as evidence of strong internal consistency while also warranting further scale refinement in future research. These results collectively support the reliability, internal consistency, and convergent validity of the measurement model, providing a solid foundation for further structural model testing.

In addition to reliability and convergent validity, standardized factor loadings were examined through confirmatory factor analysis. As reported in Supplementary Table S2, the standardized factor loadings of the measurement indicators ranged from 0.534 to 0.961, indicating acceptable item-level representation of the corresponding latent constructs. These results further support the adequacy of the measurement model. Discriminant validity was further assessed using the Fornell–Larcker criterion. As shown in Table 3, the square root of AVE for each construct was greater than its correlations with other constructs, indicating acceptable discriminant validity.

Given that all variables were collected through self-report measures at a single time point, Harman’s single-factor test was conducted to assess the potential influence of common method bias. The results showed that six factors had eigenvalues greater than 1, and the first factor accounted for 48.220% of the total variance, which is below the commonly accepted threshold of 50%. Therefore, common method bias is unlikely to pose a serious threat to the validity of the findings.

### 4.2. Structural Model Evaluation

The structural model was assessed to evaluate the hypothesized relationships among the five core constructs: social support, organizational support, AI literacy, technological self-efficacy, and AI well-being. The model demonstrated a good overall fit to the data, as evidenced by the following fit indices: χ^2^/df = 2.84, GFI = 0.92, NFI = 0.96, IFI = 0.97, TLI = 0.97, CFI = 0.97, and RMSEA = 0.06. These values are within the recommended thresholds, indicating that the hypothesized model fits the data well.

**Figure 1:**
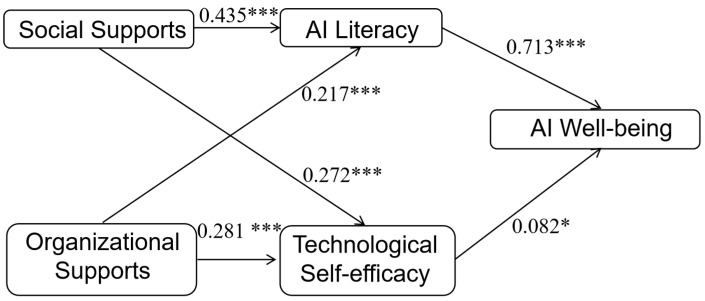
Structural equation model analysis. *Note.* * *p* < 0.05, *** *p* < 0.001.

As depicted in Figure 1 and detailed in Table 4, the results from the structural equation model provide strong empirical support for the hypothesized relationships among the five core constructs. The model reveals a coherent pathway through which external support factors (i.e., social and organizational support) shape faculty members’ psychological well-being in AI-integrated teaching contexts via two key mediators: AI literacy and technological self-efficacy.

Social support exerted a significant positive influence on both AI literacy (β = 0.435, *p* < 0.001) and technological self-efficacy (β = 0.272, *p* < 0.001), suggesting that collegial collaboration and leadership encouragement tend to be associated with higher faculty members’ AI-related competencies and confidence. Similarly, organizational support positively predicted both AI literacy (β = 0.217, *p* < 0.001) and technological self-efficacy (β = 0.281, *p* < 0.001), indicating that institutional infrastructure, policy incentives, and strategic alignment correspond to an increase in capacity building.

Among the two mediators, AI literacy emerged as the strongest predictor of AI well-being (β = 0.713, *p* < 0.001), highlighting its central role in accounting for the variance in faculty members’ positive psychological states when engaging with AI-enhanced teaching. Technological self-efficacy also showed a significant but comparatively modest effect (β = 0.082, *p* < 0.05), suggesting that while confidence in using technology predicts well-being, its influence is secondary to that of conceptual and pedagogical AI knowledge.

These findings collectively support the theorized structural model and underscore the importance of fostering both external (social and organizational) and internal (cognitive and efficacy-based) resources to improve university faculty’s adaptation to and well-being in AI-driven educational environments.

### 4.3. Mediation Analysis

To assess the stability and statistical significance of the mediated pathways in the structural model, a bootstrapping procedure was conducted with 2000 resamples using the bias-corrected percentile method to generate 95% confidence intervals. This method provides a robust estimation of indirect effects, particularly in complex mediation models involving multiple predictors and mediators. The results of the mediation analysis are presented in Table 5.

Both social support and organizational support exhibited significant indirect effects on AI well-being, mediated by AI literacy and technological self-efficacy. Specifically, the standardized indirect effect of social support on AI well-being was 0.332, while the indirect effect of organizational support was 0.178. These findings indicate that external support systems are associated with higher faculty well-being primarily by strengthening individual-level cognitive and psychological capacities rather than exerting direct emotional influence. In other words, when faculty members receive collegial encouragement and institutional resources, they are more likely to develop the knowledge, confidence, and perceived control necessary to use AI effectively, which in turn is associated with a sense of well-being in AI-integrated contexts.

Among the mediators, AI literacy accounted for the majority of the indirect effect, highlighting the central role of cognitive understanding and pedagogical competence in linking external support to positive psychological outcomes. Technological self-efficacy also served as a significant but smaller mediator, suggesting that while confidence in using AI tools predicts well-being, its effect depends on the level of AI knowledge that supports meaningful engagement. This pattern reflects a dual mediation mechanism, in which organizational and social contexts are associated with internal capacities that link AI adoption to positive psychological adaptation.

Overall, these results clarify that the path from external support to AI well-being operates primarily through the internalization of competence and confidence. The indirect effects reveal how supportive environments translate into higher AI literacy and self-efficacy, which subsequently predict educators’ greater satisfaction, reduced anxiety, and stronger professional fulfillment in AI-driven higher education settings.

## 5. Discussion

### 5.1. Theoretical Implications

Overall, our findings align closely with the integrated conceptual framework that combines social support theory and self-determination theory. From the perspective of social support theory (Cohen & Wills, 1985), both social and organizational support act as protective resources that buffer psychological strain and foster positive affective outcomes. These supports, according to self-determination theory (Deci & Ryan, 2000), are positively linked to the fulfillment of educators’ basic psychological needs for relatedness and competence, which are associated with greater intrinsic motivation and well-being.

AI literacy’s dominant role as a mediator reinforces the importance of cognitive mastery as a foundational mechanism for psychological adaptation. While self-efficacy reflects confidence in using technology, literacy embodies deeper conceptual understanding and critical judgment of AI’s pedagogical and ethical implications. Faculty who possess this literacy are better equipped to navigate technological complexity, exercise professional autonomy, and maintain a sense of control—key predictors of psychological well-being according to self-determination theory. In this sense, AI literacy serves as both a cognitive competence and an affective safeguard, reflecting how educators are able to associate uncertainty with agency and maintain stress amid innovation. Generic organizational and social support do not automatically translate into well-being; rather, AI literacy acts as the critical cognitive and ethical apparatus through which external structural support is processed and linked to ultimate well-being.

Although AI literacy showed a relatively strong relationship with AI well-being, the two constructs are theoretically distinct. AI literacy reflects faculty members’ knowledge, ethical awareness, and pedagogical capacity to use AI responsibly, whereas AI well-being captures their positive psychological functioning in AI-supported work. The strong coefficient suggests that meaningful AI understanding and pedagogical integration are closely associated with positive psychological adaptation. However, this relationship should be interpreted with caution. Given that both constructs are rooted in faculty members’ AI-related professional experiences, some degree of conceptual proximity may exist, particularly where AI literacy involves pedagogical integration and professional development, and AI well-being reflects engagement, meaning, and accomplishment. Thus, the magnitude of the association may partly result from conceptual proximity between these constructs. Nevertheless, discriminant validity results indicate that they remain empirically distinguishable in this study.

The theoretical contribution of this study lies in shifting the focus from technology adoption to AI-related psychological adaptation. Previous studies on technology acceptance have mainly emphasized perceived usefulness, ease of use, and behavioral intention, while research on teacher self-efficacy has often focused on educators’ confidence in using technology. By contrast, the present model identifies AI literacy as a broader cognitive and reflective mechanism that connects external support systems with faculty well-being. AI literacy is theoretically distinct from technological self-efficacy because it involves not only confidence in using technology but also understanding AI principles, evaluating ethical implications, and integrating AI into pedagogical practice. Therefore, the support–AI literacy–well-being pathway explains how contextual resources become internalized as meaningful professional competence, which in turn relates to positive psychological functioning in AI-integrated higher education.

### 5.2. International Contextualisation and Comparative Perspective

Although this study was conducted within the Chinese higher education system, the mechanisms identified have broader implications for global higher education. The relationship between external support, digital competence, and well-being reflects challenges that are widely shared internationally as universities move toward AI-enabled ecosystems. Reports from OECD (2023) and UNESCO (2024) similarly highlight that faculty worldwide face increased cognitive load, technostress, and ethical uncertainty when adapting to AI-driven instruction. Empirical evidence further indicates that while digital technologies are linked to flexibility, productivity, and instructional efficiency, their excessive use is associated with elevated stress levels and potential impairments in mental health and performance. Meanwhile, technostress exerts a stronger negative impact in the Asia-Pacific region than in North America, underscoring the uneven distribution of digital pressure across global educational contexts (Sharma, 2024).

This comparative insight suggests that while the pathways from support to well-being may be universal, their relative strengths are context-dependent. In highly centralized systems, institutional infrastructure and policy incentives may serve as the primary catalysts of adaptation, while in more liberal systems, interpersonal collaboration and self-regulation mechanisms could be more critical. Future cross-national research should systematically examine how cultural values (Hofstede, 2001) and governance structures moderate these relationships, advancing a more nuanced understanding of AI integration across global higher education.

### 5.3. Policy and Practical Implications

The results carry significant implications for higher education leaders and policymakers seeking to promote sustainable, human-centered AI integration. First, at the institutional level, policies should prioritize multi-layered support systems that combine infrastructural investment with emotional and professional support. Organizational initiatives should go beyond providing technology access to fostering environments that value collaboration, reflective practice, and psychological safety. Second, AI literacy development must be conceptualized as a core dimension of faculty professionalism, not merely a technical skill. Professional development programs should emphasize critical understanding, ethical reasoning, and pedagogical innovation in AI use. This aligns with UNESCO’s (2024) view that AI competency constitutes a fundamental component of teachers’ professional competence and continuous learning, emphasizing that understanding AI principles and their responsible application underpins equitable and human-centered digital transformation. Third, international collaboration in faculty AI training is increasingly essential. Universities should engage in transnational partnerships and knowledge exchanges to co-develop open-access AI training materials and culturally adaptable pedagogical frameworks. Such collaboration can bridge gaps between technologically advanced and resource-constrained contexts, promoting global inclusivity in AI-enabled education. Finally, well-being-centered AI governance should be embedded in institutional strategy. Universities must evaluate AI adoption not only by efficiency gains but also by its impact on faculty mental health, autonomy, and sense of purpose. Embedding psychological and ethical safeguards into AI policy frameworks is vital for aligning technological advancement with humanistic educational goals.

### 5.4. Limitations and Future Research

Despite its contributions, this study has several limitations. First, the cross-sectional design limits causal inference; longitudinal or experimental studies are needed to examine dynamic changes in faculty well-being over time. Second, regarding measurement instrumentation, although the adapted PERMA-based scale used to measure AI well-being demonstrated strong psychometric properties in our sample, it relies on a concise, limited number of items. Consequently, this brief operationalization may not fully capture the complex, multidimensional nature of digital well-being in its entirety. Third, although the discriminant validity results supported the empirical distinction between AI literacy and AI well-being, the strong association between these two constructs suggests a degree of conceptual proximity. Some dimensions of AI literacy, such as AI pedagogy and AI-supported professional development, may be closely related to several dimensions of AI well-being, e.g., engagement, meaning, and accomplishment. Therefore, the relatively large path coefficient from AI literacy to AI well-being should be interpreted with caution. Future studies should further refine the operational boundaries between these constructs and develop more differentiated measurement items. Fourth, the combined influence of self-report surveys that were collected from a single source at a single time point made the results susceptible to common method bias (CMB). Future research should consider gathering multi-source or multi-wave data to further mitigate this limitation. Fifth, in terms of sampling and generalizability, the data for this study were collected from a single research-intensive university in China. This specific institutional context warrants caution when generalizing the findings to other types of higher education institutions, such as teaching-oriented colleges, vocational universities, or institutions in different cultural and geographic regions, where resource allocation and institutional demands may vary. Sixth, more than half of the sample (51.79%) consisted of faculty from engineering sciences. This high concentration introduces a potential disciplinary bias, as engineering faculty may naturally possess higher baseline levels of technological self-efficacy and AI literacy than their peers in the humanities and arts, potentially skewing the results and limiting generalizability across the broader academic spectrum. Future research should employ a more balanced stratified sampling method to validate these findings across a more diverse range of disciplines. Furthermore, discipline-specific differences (e.g., between STEM and humanities faculty) merit further investigation, as pedagogical demands and AI exposure vary significantly across fields. Finally, in addition, the very high reliability coefficient of the AI well-being scale suggests that some items may be highly similar in meaning. Future studies should further examine potential item redundancy and refine the scale to ensure broader content coverage of AI well-being. Incorporating qualitative methods, such as interviews or classroom observations, could also provide deeper insights into the lived experiences of educators navigating AI integration.

## 6. Conclusions

In summary, this study demonstrates that faculty well-being in AI-integrated higher education is jointly shaped by external supports and internal psychological resources. Social and organizational support enhance well-being indirectly through the development of AI literacy and technological self-efficacy, with AI literacy playing the most pivotal role. By integrating perspectives from social support theory and self-determination theory, the study advances a multilevel framework for understanding how contextual and cognitive factors jointly foster psychological adaptation in AI-mediated environments. Beyond the Chinese context, these findings contribute to the global discourse on human-centered and sustainable AI in education, providing theoretical and policy guidance for building supportive ecosystems that empower educators to thrive in the era of intelligent teaching and learning.

## Figures and Tables

**Table 1:** Demographic characteristics of the sample.

Variable	Category	N	Percentage
Gender	Male	232	46.22%
	Female	270	53.78%
Age	Under 30	83	16.53%
	31–35	100	19.92%
	36–40	97	19.32%
	41–45	75	14.94%
	46–50	74	14.74%
	51–55	53	10.56%
	Over 56	20	3.98%
Discipline	Humanities and arts	83	16.53%
	Social sciences	137	27.29%
	Natural sciences	22	4.38%
	Engineering sciences	260	51.79%
Professional Title	Intermediate (Lecturer/Assistant Prof.)	286	56.97%
	Associate (Associate Prof.)	155	30.88%
	Senior (Professor)	61	12.15%

**Table 2:** Reliability and convergent validity of the measurement model.

Constructs	Items	KMO	Cronbach’s α	C.R.	AVE
Social Supports	8	0.837	0.951	0.881	0.715
Organizational Supports	7	0.837	0.960	0.926	0.807
AI Literacy	15	0.948	0.953	0.895	0.644
Technological Self-Efficacy	4	0.843	0.921	0.924	0.752
AI Well-Being	5	0.917	0.966	0.966	0.851

**Table 3:** Discriminant validity results.

Constructs	Social Support	Organizational Support	AI Literacy	Technological Self-Efficacy	AI Well-Being
Social Supports	0.850				
Organizational Supports	0.748	0.898			
AI Literacy	0.595	0.496	0.807		
Technological Self-Efficacy	0.492	0.449	0.501	0.867	
AI Well-Being	0.614	0.533	0.752	0.435	0.924

Note. Diagonal values represent the square roots of AVE. Off-diagonal values represent inter-construct correlations.

**Table 4:** Path coefficients and significance levels.

Hypotheses	Path	Standardized	SE	t
H1	Social support → AI literacy	0.435	0.061	7.590 ***
H2	Social support → Technological self-efficacy	0.272	0.056	4.415 ***
H3	Organizational support → AI literacy	0.217	0.064	3.895 ***
H4	Organizational support → Technological self-efficacy	0.281	0.060	4.614 ***
H5	AI literacy → AI well-being	0.713	0.039	18.390 ***
H6	Technological self-efficacy → AI well-being	0.082	0.044	2.174 *

*Note.* * *p* < 0.05, *** *p* < 0.001.

**Table 5:** Results of mediation analysis for AI well-being: direct effect, indirect effect, and total effect.

	Independent Variables	Social Support	Organizational Support	AI Literacy	Technological Self-Efficacy
Dependent Variables					
Standardized Direct Effects	AI literacy	0.435	0.217	—	—
	Technological self-efficacy	0.272	0.281	—	—
	AI well-being	—	—	0.713	0.082
Standardized Indirect Effects	AI well-being	0.332	0.178	—	—
Standardized Total Effects	AI literacy	0.435	0.217	—	—
	Technological self-efficacy	0.272	0.281	—	—
	AI well-being	0.332	0.178	0.713	0.082

## Data Availability

The data that support the findings of this study are available from the corresponding author upon reasonable request.

## Supplementary Materials

The following supporting information can be downloaded at: https://www.mdpi.com/article/10.3390/bs16071168/s1. Table S1: Survey Instruments and Scale Items; Table S2: Measurement indicators and standardized factor loadings.
