# Supplementary material for: Exploring University Faculty’s AI Well-Being: A Structural Equation Model of Social Supports, AI Literacy, and Technological Self-Efficacy
Source: Behav Sci (Basel). 2026 Jul 10;16(7):1168. doi: 10.3390/bs16071168 (PMC13405811; doi:10.3390/bs16071168)
Supplement: Supplementary file 1 [file behavsci-16-01168-s001.zip › behavsci-4263474-supplementary.pdf]

behavioral sciences

MDPI

Appendix

Supplementary Table S1. Survey Instruments and Scale Items

Construct	Item Code	Questionnaire Item
AI Literacy	LHA	1. Recognizing that AI tools are human-driven.
	LHD	2. Valuing human roles and responsibilities during analysis and decision-making in AI applications.
	LHC	3. Promoting the establishment of an inclusive human-AI collaborative society.
	LEA	4. Understanding potential ethical issues of AI technologies in education (e.g., data security, privacy).
	LED	5. Adhering to ethical norms of "safe and responsible use of AI" in teaching practices.
	LEC	6. Contributing to the resolution of ethical, socio-cultural, and environmental issues related to AI.
	LFA	7. Possessing basic knowledge of AI, including its main categories and application scenarios.
	LFD	8. Effectively applying AI in instructional design and implementation.
	LFC	9. Customizing AI tools capable of solving complex educational challenges.
	LPA	10. Identifying and utilizing the benefits of AI tools in curriculum planning, teaching, and assessment.
	LPD	11. Using AI to support differentiated learning and student-teacher interactions.
	LPC	12. Utilizing AI data and feedback to explore pedagogical innovation.
	LDA	13. Leveraging AI tools to assess personal learning needs and engage in reflective practice.
	LDD	14. Utilizing AI tools to participate in collaborative professional learning communities.
	LDC	15. Customizing AI tools to support personal career development.
AI Well-Being	WBhappy	16. Using AI makes me feel happy and satisfied in my work.
	WBfocused	17. Using AI makes me more focused and engaged when designing teaching activities.
	WBinteraction	18. AI helps me better communicate and interact with colleagues and students.
	WBmeaning	19. Integrating AI into my work allows me to experience professional meaning.
	WBachieveme	20. AI assists my work, giving me a strong sense of achievement.
Social Support	SSCST	21. I can obtain help and advice from colleagues regarding 【AI-assisted teaching】.
	SSCSR	22. I can obtain help and advice from colleagues regarding 【AI-assisted research】.
	SSCIT	23. Colleagues frequently exchange practical experiences regarding 【AI-assisted teaching】.
	SSCIR	24. Colleagues frequently exchange practical experiences regarding 【AI-assisted research】.
	SSLIT1	25. The leadership of my department/college strongly supports my involvement in 【AI-innovative teaching】 work.
	SSLIR1	26. The leadership of my department/college strongly supports my involvement in 【AI-innovative research】 work.
	SSLIT2	27. The leadership of the university and relevant offices highly values 【AI-innovative teaching】 work.
	SSLIR2	28. The leadership of the university and relevant offices highly values 【AI-innovative research】 work.
Organizational Support	OSOST	29. The university has a clear strategic plan to promote 【AI-innovative teaching】.
	OSOSI	30. The university has a clear strategic plan to promote 【AI-innovative research】.
	OSIIT	31. The university has specific incentive policies (e.g., research projects, awards, or promotion) to encourage 【AI-innovative teaching】.
	OSIII	32. The university has specific incentive policies (e.g., research projects, awards, or promotion) to encourage 【AI-innovative research】.

	OSTP	33. The university's network infrastructure and technical platforms are sufficient to support teachers in utilizing AI technologies.
	OSTT	34. The university provides relevant training or lectures on 【AI-innovative teaching】.
	OSTR	35. The university provides relevant training or lectures on 【AI-innovative research】.
Technological Self-Efficacy	PTE1	36. I frequently utilize a variety of information technologies to interact with students in my daily teaching.
	PTE2	37. If I encounter information technology problems, I can generally resolve them myself.
	PTE3	38. I am fully capable of independently using information technology to complete my daily work.
	PTE4	39. I possess rich experience in utilizing information technology.

Supplementary Table S2. Measurement indicators and standardized factor loadings.

Construct	Indicator	Description	Standardized loading
Social Support	SSCSAV	Colleague advice	0.904
Social Support	SSCIAV	Collegial interaction	0.891
Social Support	SSLIAV	Leadership support	0.747
Organizational Support	OSOSav	Strategic orientation	0.937
Organizational Support	OSIIav	Institutional incentives	0.911
Organizational Support	OSTPav	Institutional guarantees	0.845
AI Literacy	LHav	Human-centred mindset	0.534
AI Literacy	LEav	AI ethics	0.582
AI Literacy	LFav	AI foundations and applications	0.923
AI Literacy	LPav	AI pedagogy	0.961
AI Literacy	LDav	Professional development	0.926
Technological Self-Efficacy	PTE1	Technology-supported interaction	0.760
Technological Self-Efficacy	PTE2	Problem solving	0.895
Technological Self-Efficacy	PTE3	Independent use	0.880
Technological Self-Efficacy	PTE4	Technology experience	0.924
AI Well-Being	WBhappy	Positive emotion	0.864
AI Well-Being	WBfocused	Engagement	0.927
AI Well-Being	WBinteraction	Relationships	0.932
AI Well-Being	WBmeaning	Meaning	0.955
AI Well-Being	WBachievement	Accomplishment	0.936
